# Rapid Assessment of Di(2-ethylhexyl) Phthalate Migration from Consumer PVC Products

**DOI:** 10.3390/toxics12010007

**Published:** 2023-12-20

**Authors:** Jiwon An, Hyun-Ho Roh, Haeyoon Jeong, Kuen-Yong Lee, Taiyoun Rhim

**Affiliations:** 1 Department of Bioengineering, College of Engineering, Hanyang University, Seoul 04763, Republic of Korea; 2 Institute for Bioengineering and Biopharmaceutical Research, Hanyang University, Seoul 04763, Republic of Korea

**Keywords:** phthalate ester, poly(vinyl chloride), consumer product, packaging material, elution

## Abstract

Poly(vinyl chloride) (PVC) is widely used to produce various consumer goods, including food packaging, toys for children, building materials, and cosmetic products. However, despite their widespread use, phthalate plasticizers have been identified as endocrine disruptors, which cause adverse health effects, thus leading to increasing concerns regarding their migration from PVC products to the environment. This study proposed a method for rapidly measuring the migration of phthalates, particularly di(2-ethylhexyl) phthalate (DEHP), from PVC products to commonly encountered liquids. The release of DEHP under various conditions, including exposure to aqueous and organic solvents, different temperatures, and household microwaves, was investigated. The amount of DEHP released from both laboratory-produced PVC films and commercially available PVC products was measured to elucidate the potential risks associated with its real-world applications. Furthermore, tests were performed to evaluate cytotoxicity using estrogen-dependent and -independent cancer cell lines. The results revealed a dose-dependent impact on estrogen-dependent cells, thus emphasizing the potential health implications of phthalate release. This comprehensive study provides valuable insights into the migration patterns of DEHP from PVC products and forms a basis for further research on the safety of PVC and plasticizers.

## 1. Introduction

Poly(vinyl chloride) (PVC) is the most produced polymer after polyethylene and polypropylene [1]. It is widely employed to fabricate consumer products, including food and beverage packages, children’s toys, plastic bags, automobile interiors, building materials, furnishings (e.g., wallpaper, vinyl flooring, and furniture upholstery), and cosmetic products [2]. However, owing to the inherent rigidity of PVC, plasticizers are typically incorporated to confer flexibility and elasticity for specific applications [3]. More than 3 million tons of plasticizers, particularly phthalate plasticizers, are produced annually globally [4].

Despite their widespread use, PVC and its associated phthalate plasticizers have garnered considerable attention owing to their associated health and environmental risks [5]. Phthalate plasticizers, a significant component that makes PVCs flexible, have been identified as endocrine disruptors, which affect the endocannabinoid system and are directly linked to metabolic syndrome and tissue damage [6,7,8]. Di(2-ethylhexyl) phthalate (DEHP), the most commonly used phthalate ester plasticizer, interacts with estrogen receptor alpha and interferes with the normal hormonal balance, leading to estrogenic effects in the body [9]. These plasticizers can be released into the environment from various PVC products, thus posing a potential threat to human health through inhalation, ingestion, and skin contact [10,11,12,13,14,15]. Their non-covalent attachment to PVC facilitates easy migration, leading to recent efforts to explore the covalent attachment of phthalates to PVC [16].

Studies on DEHP have highlighted its adverse effects, including anti-androgenic effects at high doses (405 mg/kg/day) and subtle effects at lower doses (15 mg/kg/day) [17]. The Food and Drug Administration (FDA) further emphasizes the risks associated with oral exposure to DEHP during gestation (100–200 mg/kg/day), which include neural tube defects, skeletal and cardiovascular malformations, developmental delays, and intrauterine death [18]. However, the recent announcement by the European Food Safety Authority establishes significantly lower limits, setting a tolerable daily intake (TDI) of 50 μg/kg based on the potential for fetal testosterone depression and a TDI of 150 μg/kg based on effects on the liver [19]. DEHP is also found in PVC medical devices, subject to scrutiny from European authorities [20], resulting in strict controls over its use.

In addition to health concerns, awareness on the environmental impact of phthalate-based plasticizers originating from PVC products is increasing. Further, as the detection of microplastics in living environments has become more prevalent [21,22], the potential migration of phthalate-based plasticizers from PVC items has raised additional alarms. The harmful nature of fine plastics contributes to the complexity of this issue [23].

Despite these concerns, studies assessing the plasticizer quantity in PVC products in various living environments remain limited. Further, current methods for evaluating the plasticizer content rely on physical and chemical analyses of the melted PVC products to determine the remaining plasticizer content in the solution [24]. However, these methods lack the specificity required to ascertain whether phthalates migrate from consumer products into the body. In response to these challenges, the present study aims to overcome the lack of information by developing a method to rapidly measure the migration of phthalates from PVC products into liquid components commonly used in daily life. By subjecting phthalate-containing products to conditions encountered in living environments and assessing the amount of phthalate leaching, our aim is to identify conditions where phthalates migrate readily. This research seeks to provide a quick and straightforward method, employing simple equipment such as high-performance liquid chromatography (HPLC), to determine the extent of DEHP leaching from a PVC product in a living environment. Thus, it elucidates the potential risks associated with the use of PVC and its plasticizers in various applications.

## 2. Materials and Methods

### 2.1. Reagents and Materials

Extra-pure-grade DEHP was obtained from Samchun Chemical (Pyeongtaek, Republic of Korea). HPLC-grade *n*-hexane and glacial acetic acid were purchased from Merck (Darmstadt, Germany). Phosphate-buffered saline (PBS), Dulbecco’s modified Eagle’s medium (DMEM), RPMI 1640 medium, and fetal bovine serum (FBS) were procured from Corning Cellgro (Manassas, VA, USA). All reagents and buffer solutions were prepared in glass vials and apparatuses to prevent contamination with phthalates.

### 2.2. Preparation of Standard PVC Film

The resin suspension, sourced from Hanwha Chemical (Yeosu, Republic of Korea), was used as the base material for standard PVC film. To enhance flexibility, DEHP was incorporated into the resin at a ratio of 60 parts of DEHP per 100 parts of PVC. The resulting blend underwent a thorough melting process using a twin-screw extruder. Subsequently, the extruded resin was pelletized and washed to eliminate surface dust and impurities. Initially, granulated pellets were immersed in a 0.5% non-toxic mild soap solution and stirred thoroughly for 3 min. Following this, the pellets underwent 5 min of washing with running tap water, followed by washing with distilled water for an additional 10 min. Subsequently, the samples were treated with HPLC-grade methanol for 15 s and then dried in an oven at 50 °C for 30 min. The cleaned pellets were then shaped into a film (20 mm × 10 mm × 0.4 mm) using a steel mold operated as a hot press at 170 °C. The molded samples were promptly quenched in a water bath to room temperature. Subsequently, the samples underwent a secondary washing as described above. The molded and rinsed PVC films were then employed for leaching experiments. Furthermore, all glassware used in this study underwent thorough cleaning using a tetrahydrofuran–methanol mixture before use.

### 2.3. Migration of DEHP from PVC Films into Liquids

A two-pronged approach was adopted to investigate the release of DEHP from the PVC films. First, a PVC film produced in the laboratory and designated as the control group served as a benchmark for comparative analysis. In addition, various PVC products procured from a local market were subjected to DEHP elution tests. PVC films were cut into pieces (5 mm × 5 mm, 1 g per piece). Subsequently, various stimulants were used to facilitate DEHP release. Distilled water, saline (PBS), hydrochloric acid (pH 1), sodium hydroxide solution (pH 13), olive oil, ethanol, and acetone were used as representative solvents possibly in contact with PVC products. The samples were submerged in the stimulants (5 mL) for varying exposure times and temperatures. After the removal of the PVC samples, the solutions were preserved in glass vials for subsequent analysis.

### 2.4. High-Performance Liquid Chromatography (HPLC)

HPLC analysis was performed using a Waters HPLC system (Waters Breeze 1525, Etten-Leur, The Netherlands) equipped with a binary pump, autosampler (Waters 2707), and ultraviolet–visible detector. Chromatographic separation was achieved using a Symmetry C18 column (150 mm × 4.6 mm; particle size = 5 µm), with a mobile phase consisting of a mixture of 40% methanol and 60% acetonitrile. In each analysis, a sample volume of 10 μL was injected into the HPLC system. The flow rate was maintained at 0.6 mL/min and all eluents were monitored at 228 nm. All experiments were conducted three times, and the presented data correspond to the average of three replicates. Standard deviation is not shown due to its negligible impact.

### 2.5. Calibration Curve of DEHP for HPLC

DEHP (0.786 mg/mL) was dissolved in acetonitrile (Merck, Darmstadt, Germany) to prepare a 1000 ppm stock solution. Subsequently, the stock solution was diluted to generate a series of standard solutions of varying concentrations: 0, 50, 100, 200, and 500 ppm. A comprehensive calibration curve was constructed for all of these concentrations.

### 2.6. Cell Culture and Cytotoxicity Evaluation

MCF-7 and MDA-MB-231 cells were obtained from the Korean Cell Line Bank (Seoul, Republic of Korea). MCF-7 cells were cultured in DMEM supplemented with 5% FBS, whereas MDA-MB-231 cells were cultured in RPMI 1640 media with 10% FBS. The cells were incubated at 37 °C in a 5% CO_2_ atmosphere. Since DEHP was not soluble in media, it was initially solubilized in ethanol and then further diluted with the media. The resulting concentration of ethanol in the media was 0.1%. All of the samples were filtered through a 0.22 µm filter, and the filtered samples were introduced to MCF-7 and MDA-MB-231 cells, which had been cultured to approximately 20% confluence in 96-well tissue culture plates. After 48 h of incubation, 3-(4,5-dimethylthiazol-2-yl)-2,5-diphenyltetrazolium bromide (MTT) assay was performed according to the manufacturer’s instructions (Sigma-Aldrich, St. Louis, MO, USA). Cells were also cultured in the media only containing 0.1% ethanol and used as a control (n = 4).

### 2.7. Statistical Analysis

For group comparisons, one-way analysis of variance (ANOVA) followed by Tukey’s post hoc test using IBM SPSS version 19 was performed. Statistical significance was determined at a *p*-value less than 0.05 for all tests.

## 3. Results and Discussion

**Figure 1:**
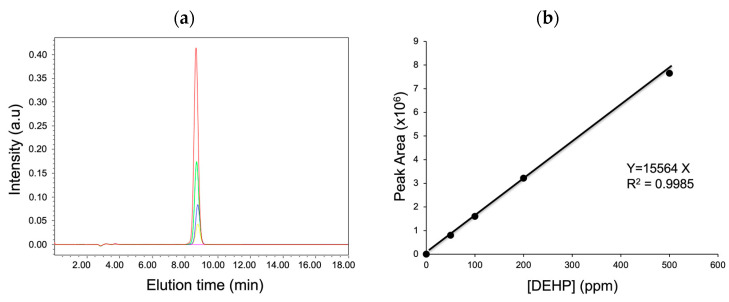
(**a**) HPLC chromatograms of DEHP standards at different concentrations (magenta, 0; yellow, 50; blue, 100; green, 200; red, 500 ppm) and (**b**) a corresponding calibration curve.

The objective of this study was to provide a standard experimental method to determine the amount of DEHP that migrated from the PVC products. Therefore, first, a calibration curve was prepared by plotting the peak area determined from the chromatogram vs. the DEHP concentration in the range of 0–500 ppm (Figure 1). Next, linear regression was performed and the correlation coefficient was determined to be 0.9985, thus suggesting a strong relationship between the peak area and DEHP concentration obtained from the HPLC analysis.

The solutions used to stimulate the release of DEHP from the PVC products were chosen based on conditions commonly used in daily life. Note that most foods contain water and/or edible oil. Additionally, acidic solutions, such as vinegar and alcoholic substances, are edible solutions used in various alcoholic beverages. Further, inedible solutions, such as alkaline solutions used in various detergents and acetone used to remove nail polish, are commonly used in living environments.

First, the amount of DEHP released from PVC films produced in the laboratory as standard samples was measured. The aforementioned aqueous solutions were applied to the PVC films under various temperature conditions (−20, 4, 25, and 37 °C), along with an extremely harsh condition realized by an autoclave (121 °C). As listed in Table 1, elution of DEHP from the PVC films was not detected in any of the collected aqueous samples, even when the films were exposed to high temperatures. This finding clearly indicates that DEHP is lipophilic [25].

By contrast, substantial amounts of DEHP were eluted into the organic solvents used depending on the experimental conditions (Table 2). In particular, regarding PVC films exposed to 90% ethanol, DEHP was not detected for 24 h; however, 4.17 ppm and 11.8 ppm DEHP were eluted from the samples after exposure for 72 h and 1 w, respectively. Exposure to 100% ethanol for 24 h did not yield detectable amounts of DEHP, whereas 11.5 ppm was detected after 72 h of exposure. Further, the DEHP release in 100% ethanol was much faster than that in 90% ethanol, thus indicating that higher ethanol concentrations yield faster DEHP release from the PVC films.

No significant DEHP release was observed when the films were exposed to olive oil for 1 w at 25 °C; however, it was detected at 121 °C, though very low compared to that exposed to ethanol. Because autoclaving is not a commonly available condition in daily life, instead of direct autoclaving, the sample was heated in olive oil using a household microwave for 15 s, which yielded a similar result to autoclaving. Interestingly, 69.0 ppm DEHP was detected after 24 h of exposure to acetone. Note that acetone tends to dissolve PVC films; thus, the experiment was performed only within 24 h to monitor the release from the films and not that from the complete dissolution of the film. Evidently, DEHP elution from the PVC films produced in the laboratory was much higher when organic solvents were used compared with when aqueous solutions were used, even under harsh conditions. Further, DEHP release was much higher with longer exposure times and higher temperatures in organic solvents.

Next, the amount of DEHP released from the PVC consumer products used in daily life was determined. Various commercially available products, including PVC packaging materials, were purchased from local markets, and the release of DEHP from these products was tested. A protective sheet is a versatile film commonly employed to safeguard surfaces, including kitchen tables and wooden furniture. Evidently, PVC products did not release DEHP when exposed to aqueous solutions at 37 °C for 24 h (Table 3). The samples heated by the microwave at 700 W for 15 s also did not significantly elute DEHP from aqueous solutions, except under strongly alkaline conditions. A protective sheet heated in the microwave released substantial amounts of DEHP under strongly alkaline conditions (pH 13). Thus, microwave exposure could be useful for the rapid testing of whether PVC products can release plasticizers under alkaline conditions. PVC products used as a protective sheet and book cover roll released substantial amounts of DEHP into olive oil, 90% ethanol, and acetone, even at 37 °C. Surprisingly, the samples heated for 15 s in the microwave exhibited increased DEHP release (Table 3). Essentially, the PVC films purchased from the local market released more plasticizers than those prepared in our laboratory. This difference could be attributed to the use of high-purity raw materials and the production of a limited quantity of film in the laboratory. It is crucial to acknowledge that the amount of plasticizer eluted may vary depending on the purity of the resin and the intricacies of the production process.

**Figure 2:**
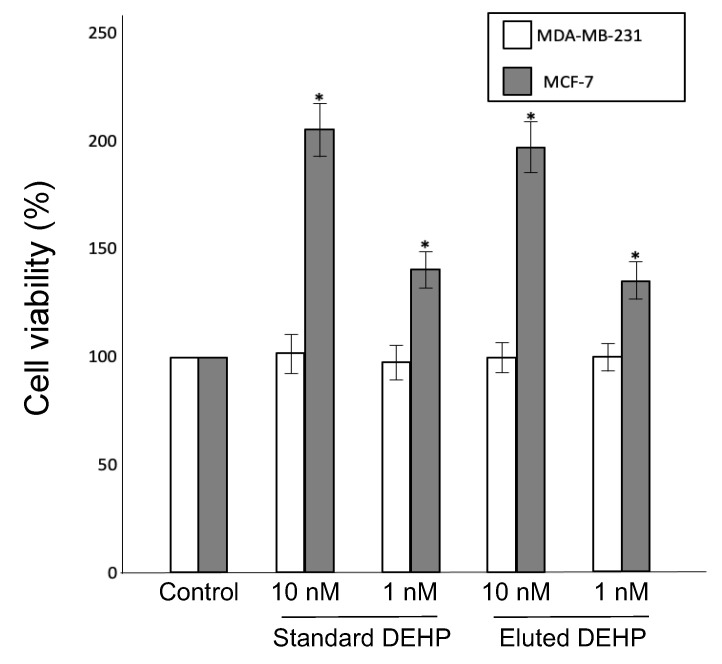
Effects of DEHP eluted from book cover rolls immersed in 90% ethanol on the viability of MCF-7 and MDA-MB-231 cells cultured for 48 h. Standard DEHP solutions were also used for comparison, and cells cultured in media only served as a control. The number of cells were counted after 48 h of culture and the cell viability was determined by comparing it to the number of control cells (mean ± standard deviation, n = 4, * *p* < 0.05 versus control).

Given that phthalate plasticizers bind to estrogen receptors and mimic estrogen action, the presence of this type of endocrine disruptor can be confirmed in estrogen-dependent and estrogen-independent cancer cell lines [9,26]. Note that MCF-7 cells are estrogen-dependent, whereas MDA-MB-231 cells are estrogen-independent [26]. In brief, the PVC products were immersed in 90% ethanol for 24 h, and an eluted plasticizer was used to test the viability of the MCF-7 and MDA-MB-231 cells. Evidently, treatment with standard DEHP or plasticizer eluted from book cover rolls did not affect the viability of estrogen-independent MDA-MB-231 cells. However, standard DEHP and the eluted plasticizer increased the proliferation of estrogen-dependent MCF-7 cells in a dose-dependent manner (Figure 2). The number of MCF-7 cells treated with the eluted DEHP was increased by 1.3 and 1.9 times for concentrations of 1 nM and 10 nM, respectively, compared to the control group.

Plasticizers are the most popular plastic additives for enhancing the flexibility and processability of materials; in particular, approximately 90% of them are used in PVC applications [27]. Despite being integral to PVC production globally, phthalate plasticizers face legal restrictions in toys and food packaging in numerous countries owing to heightened environmental awareness and growing social pressure. Thus, alternative plasticizers that meet environmental criteria without compromising the end properties of the products must be developed [28]. This study revealed a notable discrepancy in the amount of plasticizer eluted from consumer PVC products sourced from local markets compared with PVC films fabricated in the laboratory. This variance underscores the potential impact of resin purity and production process on plasticizer release. The methodological approach employed herein enabled the swift and thorough exploration of DEHP migration under diverse conditions, thus offering insights into the complexities of plasticizer release from PVC products.

## 4. Conclusions

In this study, the migration of phthalates, specifically DEHP, from PVC products was found to depend on various environmental conditions. A comprehensive evaluation of the laboratory-produced PVC films and commercially available PVC products revealed distinct patterns of DEHP release, thus emphasizing the role of exposure time, temperature, and solvent type in the migration process. Importantly, the potential health risks associated with phthalate release, particularly in estrogen-dependent cell lines, were highlighted. The methodology reported herein provides a rapid and effective means of assessing DEHP migration under diverse conditions, thus offering insights into plasticizer release from consumer PVC products compared with laboratory-produced films. These findings contribute to the evaluation of the safety of PVC and its plasticizers, essentially highlighting the variability in plasticizer release depending on the source and production process of PVC products. As regulatory scrutiny of phthalates intensifies, this study may provide valuable information to consumers regarding the potential risks associated with the use of PVC in everyday products.

## Figures and Tables

**Table 1:** Amounts of DEHP released from PVC films produced in the laboratory and treated with aqueous solutions under various temperatures and times.

Stimulant	Exposure Temperature (°C)	Exposure Time	Concentration (ppm)
distilled water	−20	4 weeks	ND ^a^
	4	4 weeks	ND
	25	4 weeks	ND
	37	4 weeks	ND
	121 ^b^	30 min	ND
PBS	−20	4 weeks	ND
	4	4 weeks	ND
	25	4 weeks	ND
	37	4 weeks	ND
	121	30 min	ND
pH 1	−20	4 weeks	ND
	4	4 weeks	ND
	25	4 weeks	ND
	37	4 weeks	ND
	121	30 min	ND
pH 13	−20	4 weeks	ND
	4	4 weeks	ND
	25	4 weeks	ND
	37	4 weeks	ND
	121	30 min	ND

^a^ Not detected (below detection limit). ^b^ Autoclaved.

**Table 2:** Amounts of DEHP released from PVC films produced in the laboratory and treated with organic solvents under various conditions of temperature and time.

Stimulant	Exposure Temperature (°C)	Exposure Time	Concentration (ppm)
90% ethanol	37	24 h	ND ^a^
	37	72 h	4.17
	37	1 week	11.8
	121 ^b^	30 min	11.3
	M ^c^	15 s	12.1
100% ethanol	37	24 h	ND
	37	72 h	11.5
olive oil	37	24 h	ND
	37	72 h	ND
	37	1 week	ND
	121 ^b^	30 min	0.35
	M ^c^	15 s	0.37
acetone	37	24 h	69.0

^a^ Not detected (below detection limit). ^b^ Autoclaved. ^c^ Microwave used (700 W).

**Table 3:** Amounts of DEHP released from various consumer PVC products depending on various stimulants and treatment conditions (unit: ppm).

	Stimulant	Condition	Distilled Water	pH 1	pH 13	Olive Oil	Ethanol (90%)	Acetone
Product								
protective sheet		37 °C, 24 h	ND ^b^	ND	ND	11261	1022	34,156 ^c^
		M ^a^, 15 s	ND	ND	18.1	33,885	10,429	15,386 ^c^
cover roll		37 °C, 24 h	ND	ND	ND	9294	8648	26,878 ^c^
		M, 15 s	ND	ND	ND	18,398	32,824	12,636 ^c^
adhesive sheet		37 °C, 24 h	ND	ND	ND	ND	ND	4.3
		M, 15 s	ND	ND	ND	4.8	9.7	10.3
hand warmer		37 °C, 24 h	ND	ND	ND	1239	927	3028 ^c^
		M, 15 s	ND	ND	ND	1528	1402	8735 ^c^

^a^ Microwave used (700 W). ^b^ Not detected (below detection limit). ^c^ Products were partially melted.

## Data Availability

Data are contained within the article.
